# Applications of functional near-infrared spectroscopy in non-drug therapy of traditional Chinese medicine: a review

**DOI:** 10.3389/fnins.2023.1329738

**Published:** 2024-01-24

**Authors:** Shifang Fu, Fanqi Liu, Xiaoyu Zhi, Yu Wang, Yijia Liu, Hao Chen, Yanguo Wang, Mingchi Luo

**Affiliations:** ^1^Traditional Chinese Medicine Rehabilitation Center, Second Affiliated Hospital of Tianjin University of Traditional Chinese Medicine, Tianjin, China; ^2^Department of Graduate School, Tianjin University of Traditional Chinese Medicine, Tianjin, China

**Keywords:** non-drug therapy of traditional Chinese medicine, fNIRS, acupuncture, massage, tai chi chuan, Baduanjin

## Abstract

Non-drug therapies of traditional Chinese medicine (TCM), including acupuncture, massage, tai chi chuan, and Baduanjin, have emerged as widespread interventions for the treatment of various diseases in clinical practice. In recent years, preliminary studies on the mechanisms of non-drug therapies of TCM have been mostly based on functional near-infrared spectroscopy (fNIRS) technology. FNIRS is an innovative, non-invasive tool to monitor hemodynamic changes in the cerebral cortex. Our review included clinical research conducted over the last 10 years, establishing fNIRS as a reliable and stable neuroimaging technique. This review explores new applications of this technology in the field of neuroscience. First, we summarize the working principles of fNIRS. We then present preventive research on the use of fNIRS in healthy individuals and therapeutic research on patients undergoing non-drug therapies of TCM. Finally, we emphasize the potential for encouraging future advancements in fNIRS studies to establish a theoretical framework for research in related fields.

## Graphical Abstract

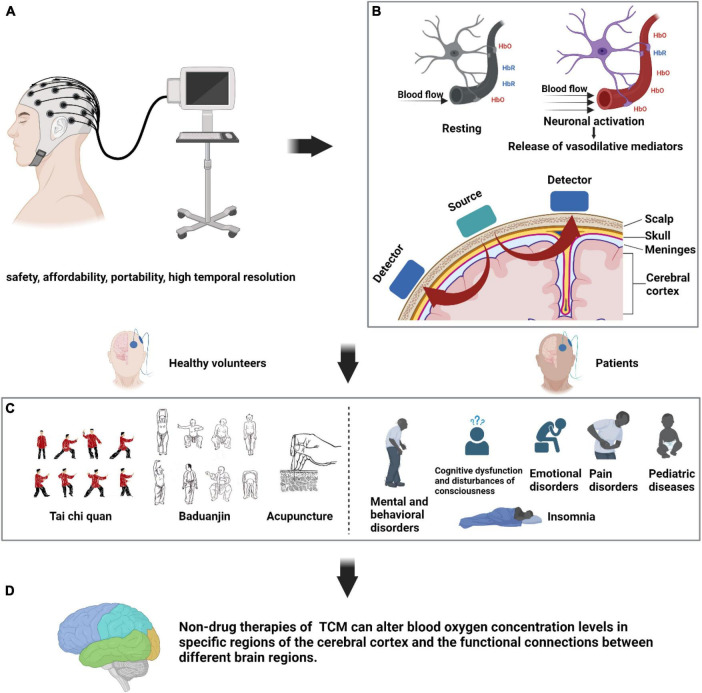

## 1 Introduction

Non-drug therapies of TCM, such as acupuncture, massage, tai chi chuan, and Baduanjin, are based on the theory of meridians and viscera in TCM. The advantages of TCM through body adjustments, heart adjustments, and breathing regulation, include regulating motor function, balance, coordination ability, and overall disease resistance and rehabilitation ability of patients at multiple levels and angles (Guo et al., 2023). TCM characteristics also include fewer adverse reactions and high clinical acceptance, which are widely used in the prevention and treatment of diseases (Li et al., 2018). Therefore, an increasing number of studies have focused on non-drug therapies of TCM. Although the scientific mechanisms of non-drug therapies of TCM remain unclear, recent studies have demonstrated that acupuncture, massage, tai chi chuan, and Baduanjin cause interactions within

the nervous system, especially regarding neuronal response regulation in the human brain (Chen Y. F. et al., 2022; Wang et al., 2022; Xu L. P. et al., 2022; Yao et al., 2022; Chen L. et al., 2023). However, the human brain is the least accessible organ; therefore, *in vivo* studies of the human brain rely predominantly on neuroimaging. Traditional brain imaging techniques include functional magnetic resonance imaging (fMRI) (Zhu et al., 2015) and electroencephalography (EEG) (Yu et al., 2019). fMRI uses magnetic fields to measure responses that depend on the blood oxygen level, a proxy for brain activity, in cortical and subcortical brain regions. Its ability to image subcortical regions and high spatial resolution has made fMRI the neuroimaging method of choice for investigating brain function in pediatric populations. fMRI is the gold standard for *in vivo* imaging of the human brain (Gossé et al., 2022). However, fMRI has a poor temporal resolution, high sensitivity to motion artifacts, and is relatively expensive. EEG and fMRI are complementary neuroimaging techniques. fMRI measures changes in blood flow, whereas EEG measures changes in electrical charges in the brain (Keren et al., 2018). Although EEG has fewer limitations than fMRI, its spatial positioning is poor. These limitations make the widespread application of fMRI and EEG in the study of the underlying brain mechanisms of non-drug therapies of TCM unappealing.

Functional near-infrared spectroscopy (fNIRS) uses near-infrared light to monitor brain activity (Pinti et al., 2020). Compared to traditional functional brain imaging technologies, fNIRS is less susceptible to noise and requires less restriction of head or body activity than MRI and computed tomography (Wilcox and Biondi, 2015). Although both fNIRS and fMRI measure alterations in blood flow in the brain, fNIRS has contributed significantly to the field of neuroscience by studying real-life scenarios, high-workload environments, and face-to-face interactions that cannot be evaluated using fMRI (Hirsch et al., 2021). fNIRS is highly versatile, can be used simultaneously with electrophysiological signal monitoring equipment, has better spatial resolution than EEG, and can potentially be used in natural environments (Llana et al., 2022). fNIRS is also insensitive to the movement of study participants; therefore, motion interference in its signals is much smaller than that in electrical signals, making it more suitable for application in natural environments (Quaresima and Ferrari, 2016). In addition, fNIRS is relatively inexpensive and can be used on a large scale. Due to frequent repeated measurements, fNIRS can be easily employed in longitudinal studies, which are becoming increasingly important for investigating the development and treatment of limb movements, mood, cognitive function, and consciousness disorders (Jang et al., 2020; Zhang et al., 2021; Khan et al., 2022; Liu et al., 2022).

Using fNIRS for assessing cerebral blood flow (CBF) changes during neuronal activation gradually increased in the mid-80s (Csipo et al., 2019). fNIRS possesses potential for widespread application in the study of non-drug therapies of TCM owing to its safety, affordability, portability, and high temporal resolution. Although several fNIRS studies on TCM applications have been conducted, to the best of our knowledge, no review article has explored most TCM applications using fNIRS. Therefore, we present a review of TCM applications of fNIRS and highlight the future perspectives of this modality and its clinical applications (Graphical Abstract).

## 2 Basic principle of fNIRS

Upon receiving stimulation from the internal body or external environment, neural activity in certain brain regions is enhanced, leading to increased oxygen consumption and changes in the concentrations of oxygenated hemoglobin (oxy-Hb, HbO_2_) and deoxyhemoglobin (deoxy-Hb, dHb) in the blood vessels. This triggers a range of brain regulatory activities including local vasodilation and increased CBF (Claassen et al., 2021). This process is known as neurovascular coupling (Liao et al., 2013; Herold et al., 2018). Changes in HbO_2_ and dHb concentrations in the blood form the basis for fNIRS testing.

Brain tissue is relatively transparent to NIRS; fNIRS is performed using near-infrared light with a wavelength of 700–900 nm in the medical field. HbO_2_ and dHb, the main chromophores in the near-infrared spectrum of blood, can absorb near-infrared light and cause light intensity attenuation; however, their absorption coefficients differ (Jöbsis, 1977; Almajidy et al., 2020). During fNIRS detection, the light source emitter emits photons and projects them onto the scalp of the participant, which then passes through the skull into the upper cortical region. Some of the photons are absorbed by HbO_2_ and dHb, while the unabsorbed photons are scattered, reflected, and return to the scalp surface along an elliptical path to be received by the detector of the fNIRS device (Rupawala et al., 2018; Paulmurugan et al., 2021). According to the modified Bier–Lambert law, the number of chromophore groups can be estimated by the attenuation of photons, that is, the alterations in HbO_2_ and dHb concentrations can be specifically calculated, particularly the increase in HbO_2_ concentration, which is an indirect reflection of the neural activity (Soekadar et al., 2021). fNIRS signals are more sensitive to microvessels with diameters of < 1 mm (Ferrari and Quaresima, 2012). Photon absorption increases with an increase in the vascular diameter, thus resulting in a decrease or even disappearance of light signals received by the scalp surface and eventually decreasing in the sensitivity of fNIRS detection (Ferrari and Quaresima, 2012). The fNIRS data contain noise from various sources, mainly physiological processes (respiration and heartbeat), head movement, and device factors, which may mask or confuse task-related cortical functional signals. Therefore, it is necessary to preprocess the collected fNIRS data and conduct a statistical analysis. Relevant studies have reviewed these methods (Tak and Ye, 2014; Kamran et al., 2016; Chen W. L. et al., 2020; Dans et al., 2021).

Functional near-infrared spectroscopy reveals the brain effects of non-drug therapies of TCM, which have been applied for the treatment of cognitive disorders, depression, anxiety, limb movement disorders, pain, attention deficit/hyperactivity disorder, and myopia in children. Clinical research using fNIRS requires a specific data acquisition scheme, which has two main design paradigms, namely task-based and resting-state-based. In task-based research, participants perform certain tasks or receive stimuli during the fNIRS data collection. The type of task or stimulus presentation has a block or event-related design. Most resting-state designs include longitudinal studies on treatment of a specific condition, with fNIRS data collected before and after the intervention. Acupuncture, massage, tai chi chuan, and Baduanjin are non-drug therapies of TCM, which have obvious TCM characteristics; thus, appropriate treatment methods should be adapted for designing the data-acquisition scheme for fNIRS detection. For example, acupuncture and massage operations performed by doctors have a short duration, the task method can be designed to observe brain activation areas and a resting state to observe the functional connectivity (FC) between the regions of interest. However, the tai chi chuan and Baduanjin exercises have a longer single-exercise duration, rendering the resting-state design more suitable.

## 3 Review methods

Studies were identified by a comprehensive search of the following databases: PubMed, Web of Science, China National Knowledge Infrastructure (CNKI), Wan Fang database and Chinese Scientific Journal Database (VIP). The search was conducted between January 1, 2014, and September 30, 2023. The search terms in the English and Chinese literature were (acupuncture OR tuina OR massage OR moxibustion OR guasha OR cupping OR Tai Chi OR Baduanjin OR wuqinxi OR yijinjing OR TCM Five Element Music Therapy) and (fNIRS OR Functional Near-Infrared Spectroscopy).

## 4 Results

After searching for keywords, 46 articles about non-drug therapies of TCM and fNIRS were selected, of which 40 were conducted in China and 6 in foreign countries. There were 12 studies on traditional sports, 32 on acupuncture and 2 on massage (Tables 1, 2).

**TABLE 1:** fNIRS research on health participants.

References (Region)	Year	Characteristics of participants		Type of intervention		fNIRS parameters	fNIRS paradigm	Brain region	Main findings
		**Experimental group**	**Control group**	**Experimental group**	**Control group**				
**Tai chi chuan**									
Chen Y. et al., 2022 (Jinan, China)	2022	Healthy older adults *n* = 18	Healthy older adults *n* = 18	Practiced Tai Chi for 16 weeks, 4 times per week	Received health lectures for 16 weeks, 4 times per week	22-channels; 13.3 Hz	Performed the NOT and negotiating obstacle with the cognitive task (NOCT) at their preferred speed along a 6m walkway	BA10	After intervention in the tai chi chuan group under NOCT, ΔHbO_2_ in right BA10 was significantly greater
Wang et al., 2022 (Beijing, China)	2022	Professional tai chi chuan athletes *n* = 13 (7M, 6F) Age: 22.8 ± 1.64y	Beginners in tai chi chuan *n* = 11 (6M, 5F) Age: 22.3 ± 1.12y	/	/	48-channels; 10Hz	Resting-state for 5 min; Chen style Tai Chi for 5 min	PFC, SMC	In the resting state, the FC between the LPFC and the RSMC in the expert group was lower than novice group. In the exercise state, the FC between the LPFC and RSMC, the RPFC and LSMC, and the LSMA and RSMC in the expert group were lower than novice group
Xie, 2022 (Jiangxi, China)	2022	Healthy elderly people *n* = 24 (9M, 15F); 1 year of practice (*n* = 6, 56.83 ± 8.11y), 3–5 years of practice (*n* = 7, 65 ± 7.65y), 10 years of practice (*n* = 11, 67 ± 11.09y)	Healthy elderly people *n* = 7 (3M, 4F); Age: 60.29 ± 8.44y	Regular exercise, 3 times/week	No regular exercise	20-channels; 7.8 Hz	Block design; Go/NO Go task	PFC	Highest HbO_2_ concentration in individuals had practiced for 3–5 years and practiced for 10 years. Mainly activating the dPFC and frontal polar region
Chen W. et al., 2022 (Jinan, China)	2022	Hong chuan tai chi chuan practitioners *n* = 18 (10M, 8F) Age: 55.78 ± 2.64 y	Tai Chi-native healthy controls *n* = 22 (13M, 9F) Age: 54.69 ± 3.10y	/	/	14-channels; 10 Hz	Resting-state design; 15 min	PFC, MC, OC	Hong chuan tai chi chuan practitioners showed higher of the PFC, MC, and OC in the resting state within the same brain hemispheres or between the left and right hemispheres
Chen et al., 2021 (Shanghai, China)	2021	Healthy young individuals *n* = 32 (12M, 20F) Age: 22 ± 1 y	/	/	/	10 Hz	Two TC one-leg stance postures, right heel kick (RHK) and left lower body and stand on one leg (LSOL), and two yoga postures, one-leg balance and Tree	PMC, SMA, dPFC	HbO_2_ changes in SMA were higher during RHK, LSOL, and Tree than OLS. The right dPFC activation was significantly greater during the RHK than during the Tree, OLB, and OLS
Yang et al., 2020 (Beijing, China)	2020	Healthy elderly people *n* = 18 (18F) Age: 66.31 ± 4.25y	Healthy elderly people *n* = 18 (18F) Age: 65.92 ± 3.48y	8-week tai chi chuan intervention (45 min per session for 3 days per week)	General daily activities.	44-channels	Block design; Flanker task	PFC, MC, OC	Increase HbO_2_ in PFC during the incongruent flankers after the tai chi chuan exercise intervention
Xie et al., 2019 (Beijing, China)	2019	Chen style tai chi chuan practitioners *n* = 23 (12M, 11F) Age: 65.01 ± 2.61 y	Healthy controls *n* = 32 (17M, 15F) Age: 65.34 ± 2.97y	/	/	14-channels; 10Hz	Resting-state for 15 min; Chen style tai chi chuan for 15 min	PFC, MC, OC	Significant differences in brain activity and dynamic configuration of connectivity were observed between the Chen style tai chi chuan group and the control group during resting and movement states
Yang, 2019 (Beijing, China)	2019	Healthy elderly people *n* = 13 (3M, 10F); Age: 66.31 ± 4.25 y	Healthy elderly people *n* = 13 (3M, 10F); Age: 65.92 ± 3.48y	24 style tai chi chuan, 3 times/week, 8 weeks	Regular physical activity	44-channels;	Block design; N-back task, Flanker task	Frontal lobe	Increase HbO_2_ in frontal lobe during Flanker task and N-back task
**Baduanjin**									
Yao et al., 2022 (Jinan, China)	2022	Baduanjin imagery group *n* = 24 Age: 67.67 ± 4.92y Baduanjin exercise group *n* = 25 Age: 67.67 ± 4.92y	Healthy controls *n* = 23 Age: 66.35 ± 4.89y	Baduanjin imagery training; the Baduanjin exercise	Reading of neutral material	19-channels; 10Hz	Block design; stroop task	PFC	Significant interaction effect between the test phase and group in the left dPFC. The HbO_2_ variations were significantly higher in participants of the Baduanjin imagery group and Baduanjin exercise group than in the control group
Li, 2020 (Guangzhou, China)	2020	Healthy college students *n* = 20 Age: 66.72 ± 4.61y	Healthy college students *n* = 23 Age: 65.86 ± 4.35y	Practiced Baduanjin for 12 weeks, 3 times per week	General daily activities for 12 weeks.	19-channels; 10Hz	Block design; Stroop task	Bilateral dPFC, vPFC, FPA	There is a correlation between the left dorsolateral prefrontal cortex and the improvement of inhibitory control ability in college students through Baduanjin exercise intervention
Jiang et al., 2019 (Beijing, China)	2019	Baduanjin practitioners *n* = 14 Age: 65.86 ± 4.35y	Baduanjin-native healthy controls *n* = 15 Age: 64.73 ± 4.68y	/	/	22-channels; 10Hz	Block design; verbal fluency test (VFT), respectively	Frontal lobe, OC	Increase HbO_2_ in bilateral PFC in Baduanjin practitioners
**Acupuncture**									
Song et al., 2023 (Tianjin, China)	2023	Healthy volunteers *n* = 29 (12M, 17F) Age: 25.03 ± 0.66y	/	Acupuncture Shousanli	/	46-channels; 11Hz	Block design; acupuncture	Frontal, parietal, and temporal cortex	Acupuncture mainly activated bilateral PFC, *deqi* acupuncture enhanced the functional connection between the frontal, parietal and temporal lobes
Cao et al., 2023 (Chengdu, China)	2023	Healthy participants *n* = 31 Age: 20.70 ± 2.34y	/	Acupuncture Quchi using three different reinforcing and reducing methods	/	36-channels; 3.096Hz	Resting-state 5 min Acupuncture Quchi 2 min 10 s	PFC, bilateral somatosensory cortex	The even reinforcing-reducing manipulation deactivated the bilateral dPFC, FPA, the right M1, the bilateral S1, and the bilateral S2; the reducing manipulation deactivated the bilateral dPFC; the reinforcing manipulation activated the bilateral dPFC, the left S1, and the right S2
Qu et al., 2023 (Chengdu, China)	2023	Healthy participants *n* = 33 Age: 20.70 ± 2.34y	/	Shaoshanhuo reinforcing method (SSH) and Toutianliang reducing method (TTL) on Quchi	/	36-channels; 3.91Hz	Block design; SSH and TTL	SMC, PFC	SSH could activate bilateral M1 and left S1. As Toutianliang reducing method, bilateral prefrontal lobe and bilateral PMC were activated. The FC increased between the left prefrontal lobe and the PMC, but there was no significant difference between the two acupuncture methods
Chen L. et al., 2023 (Chengdu, China)	2022	Healthy participants *n* = 31 (all male) Age: 20.71 ± 2.34y	Healthy participants *n* = 33 (all male) Age: 19.97 ± 1.36y	Acupuncture at Quchi	Gently tapped the skin over the Quchi	36-channels; 3.91Hz	Resting-state 5 min Acupuncture or stimulation 2 min	SMC, PFC	Inter-brain neural synchronization (INS) in the PFC of “patient”–acupuncturist dyad was significantly increased during verum but not sham acupuncture stimuli, and positively correlated with the needling sensations of “patients”
Yuan et al., 2021 (Xi’an, China)	2021	Healthy volunteers *n* = 16 (7M, 9F) Age: 25.03 ± 0.66y	/	Low-Frequency magnetic stimulation of Shenmen point	/	46-channels; 100Hz	Resting-state 2 min; Stimulate Shenmen 2 min	PFC	Compared with the resting state, the mean and integral values of HbO_2_ concentration were decreased during the task period, recovery period, and the whole process in the magnetic stimulation
Si et al., 2021 (Tianjin, China)	2021	Healthy volunteers *n* = 20 (12M, 8F) Age: 23.9 ± 1.5y	/	Acupuncture Hegu	/	48-channels; 9Hz	Quasi-experimental design	PFC, MC, BA8	The bilateral PFC and MC were significantly inhibited during acupuncture manipulation, evidenced by the decreased power of HbO_2_. The network connections with bilateral PFC showed increased. The network’s efficiency improved by acupuncture manipulation
Fernandez Rojas et al., 2019 (Canberra, Australia)	2019	Healthy volunteers *n* = 11 (12M, 8F) Age: 23.9 ± 1.5y	/	Acupuncture Hegu	/	24-channels; 10Hz	Block design; acupuncture	C3, C4 in somato-sensory region	The ΔHbO_2_ and FC of the statistical analysis showed significant differences between needle insertion, needle twisting and needle remove in very-low frequency oscillations and low frequency oscillations.

M, male; F, female; FC, functional connectivity; PFC, prefrontal cortex; dPFC, dorsolateral prefrontal cortex; vPFC, ventrolateral prefrontal cortex; FPA, frontal area; MC, motor cortex; OC, occipital cortex; SMC, sensorimotor cortex; BA, brodmann area; S1 ± primary somatosensory cortex; M1 ± primary motor cortex; S2 ± secondary somatosensory cortex.

**TABLE 2:** fNIRS research on patients.

References (Region)	Year	Characteristics of participants		Type of intervention		fNIRS Parameters	fNIRS paradigm	Brain region	Main findings
		**Experimental group**	**Control group**	**Experimental group**	**Control group**				
**Cognitive dysfunction and disturbance of consciousness**									
Khan et al., 2022 (Busan, Korea)	2022	MCI patients *n* = 11 (0M, 11F); Age: 61.58 ± 6.55y	Healthy people *n* = 11 (0M, 11F); Age: 55.92 ± 7.65y	Acupuncture therapy	/	20-channels; 7.81 Hz	Block design; working-memory task	PFC	Acupuncture therapy improved the hemodynamic responses of the patients. And the activated area of the MCI patients, as well as the connectivity, increased with acupuncture
Jeong et al., 2021 (Daejeon, Korea)	2021	MCI patients *n* = 22; Age: 50–75y	MCI patients *n* = 22; Age: 50–75y	Neurofeedback-acupuncture combined treatment	Acupuncture treatment	/	Block design; delayed matching to sample task	PFC	/
Chen J. et al., 2020 (Hangzhou, China)	2020	PSCI patients *n* = 28 (16M, 12F); Age: 65.3 ± 6.8y	PSCI patients *n* = 28 (17M, 11F); Age: 64.5 ± 7.2y	Scalp acupuncture with cluster needling++drug treatment	Drug treatment	12-channels; 10 Hz	Resting-state design	Frontal lobe, parietal lobe movement area	Applying cluster needling of scalp acupuncture on top of drug treatment can significantly elevate the cerebral hemoglobin levels compared to patients treated with drug only
Sun, 2019 (Shenyang, China)	2019	PSCI patients *n* = 30 (21M, 9F); Age: 55.50 ± 10.38y	PSCI patients *n* = 32 (22M, 10F); Age: 58.34 ± 9.51y	Scalp acupuncture treatment+Routine rehabilitation training and drug treatment	Routine rehabilitation training and drug treatment	16-channels; 10 Hz	Task state design; resting-needling the scalp while retaining the needle-removing the needle	PFC	The statistics of changes in HbO_2_ concentration in the PFC under acupuncture state are higher than those under resting state
Ghafoor et al., 2019 (Busan, Korea)	2019	MCI patients *n* = 12 (0M, 12F); Age: 61.58 ± 6.55y	Healthy people *n* = 12 (0M, 12F); Age: 55.92 ± 7.65y	Acupuncture therapy	/	20-channels; 7.81 Hz	Block design; working memory task	PFC	The t-maps of MCI were enhanced. Furthermore, an increased FC in the PFC in MCI cases in comparison to before acupuncture was obtained, and an increasing trend in the graph theory parameters was observed
Eun-Sun et al., 2018 (Daejeon, Korea)	2018	MCI patients *n* = 12; Age: 40–80y	Healthy people *n* = 12; Age: 40–80y	Acupuncture treatment	/	20-channels; 7.81 Hz	Block design; working memory task	PFC	/
**Motor dysfunction**									
Zhang et al., 2023 (Guangzhou, China)	2023	Patients with stroke hemiplegia *n* = 20 (5M, 15F); Age: 55.95 ± 16.98y	Patients with stroke hemiplegia *n* = 20 (10M, 10F); Age: 59.8 ± 11.63y	Auricular acupuncture; therapy+regular rehabilitation training; 5 times/week, 2 weeks	Sham auricular acupuncture+regular rehabilitation training 5 times/week, 2 weeks	38-channels; 10Hz	Resting-state design; 10 min	M1	Auricular acupuncture has an ameliorative effect on upper limb motor deficits after stroke and that activation of the M1 region of the brain may be a key node in auricular acupuncture for treating upper limb dysfunction in stroke patients
Chen Y. F. et al., 2023 (Shanghai, China)	2023	Upper limb motor dysfunction after stroke *n* = 10 (8M, 2F); Age: 58.00 ± 10.31y	Healthy subjects *n* = 8 (5M, 3F); Age: 49.25 ± 6.82y	Tui Na: one-finger Zen manipulation at Hegu	Tui Na: one-finger Zen manipulation at Hegu	21-channels; 19Hz	Block design: six cycles: rest (20 sec); Tui Na (20 sec); rest (30 sec)	PMC, M1, SMA, SAC	Hemodynamics of contralateral SM1 was obviously enhanced, but there was no similar pattern in ipsilateral SM1; significant difference between lateralization index values for the affected arm and the less affected arm
Chen Y. F. et al., 2022 (Shanghai, China)	2022	Patients with stroke hemiplegia *n* = 45	Patients with stroke hemiplegia *n* = 45	rTMS + Tui Na + conventional rehabilitation	rTMS + conventional rehabilitation	32-channels; 19Hz	Resting-state: 8 min; Block design: six cycles: rest (10 sec); hand movements (15 sec); rest (20 sec)	M1, PMC, SMA	/
Tang et al., 2022 (Harbin, China)	2022	Patients with lower extremity motor dysfunction after stroke *n* = 24, randomly divided into rehabilitation group *n* = 12 (11M, 1F) (Age: 51.91 ± 4.63y) and acupuncture-rehabilitation group *n* = 12 (11M, 1F) (Age: 53.50 ± 6.00y)	Healthy people *n* = 10 (9M, 1F); Age: 46.10 ± 11.00y	Rehabilitation group: routine rehabilitation training; acupuncture-rehabilitation group: acupuncture-rehabilitation intervention; 5 times/week, 4 weeks	/	24-channels; 10Hz	Walking task	SMA, PMC, SMC	Acupuncture with rehabilitation therapy can significantly improve the lower limb motor function and asymmetrical activation of SMC in stroke patients. The recovery of lower limb motor function may be related to the enhanced activation of affected PMC
Luo et al., 2022 (Mianyang, China)	2022	Observation group: patients with cerebral infarction *n* = 43 (29M, 14F) (Age: 65.77 ± 5.36y) Motor imagination group: patients with cerebral infarction *n* = 43 (30M, 13F) (Age: 66.42 ± 6.32y)	Patients with cerebral infarction *n* = 43 (27M, 16F); Age: 64.94 ± 6.86y	Observation group: basic medicines and routine rehabilitation+ exercise imaginary tai chi chuan steps Motor imagination group: basic medicines and routine rehabilitation+ exercise imaging therapy 5 times/week, 8 weeks	Basic medicines and routine rehabilitation 5 times/week, 8 weeks	22-channels	Resting-state design	SMA, PMC, SMC	Tai chi chuan imagination therapy can effectively improve the movement, balance function and abnormal gait of patients’ lower limbs, and improve the ability of daily living of patients
Jang et al., 2020 (Daejeon, Korea)	2020	Parkinson’s patients *n* = 13 (10M, 3F); Age: 65.38 ± 7.81y	Parkinson’s patients *n* = 13 (7M, 6F); Age: 61.46 ± 8.33y	Acupuncture twice a week for 4 weeks+ conventional therapy	Conventional therapy 4 weeks	4.17Hz	Task-state design	S1, M1, SMA, PFC, PMC	Acupuncture as an adjunct treatment can improve low-dose gait in PD patients and can improve balance, including activation of the cerebral cortex (PFC and auxiliary motor areas)
**Mental and behavioral disorders**									
Zhang et al., 2021 (Shenzhen, China)	2021	Major depressive disorder patients *n* = 47 (10M, 37F); Age: 39.7 ± 12.24y	/	Acupuncture therapy	/	52-channels; 10Hz	Task state design; Verbal fluency task	PFC	A single session of acupuncture demonstrated a tendency to enhance the activation of the PFC in patients with severe depression
Wong et al., 2021 (Hong Kong, China)	2021	Depressed patients *n* = 8; Age: 44.8 ± 10.3y	Depressed patients *n* = 12; Age: 50.9 ± 11.1y	Acupuncture therapy+drug treatment	Drug treatment	18-channels; 7.81Hz	Resting-state design	dPFC	Depressed patients receiving acupuncture combined with antidepressants have the stronger rsFC in the dPFC compared to those using antidepressants alone
Xiang, 2017 (Chengdu, China)	2017	Generalized anxiety disorder patients *n* = 12 (2M, 10F); Age: 45.42 ± 13.41y	Healthy people *n* = 12 (3M, 9F); Age: 40.25 ± 11.99y	Acupuncture therapy	/	250Hz	Block design; Acupuncture stimulation-Acupuncture preparation-Acupuncture stimulation	dPFC	Acupuncture can activate the left dPFC in patients with generalized anxiety disorder, resulting in an immediate response characterized by an increase in HbO2 and a decrease in dHb
Miao, 2016 (Chengdu, China)	2016	Depressed patients *n* = 8 (3M, 5F); Age: 47.62 ± 6.72y	Depressed patients *n* = 8 (2M, 6F); Age: 48.12 ± 7.74y	Acupuncture therapy	Drug treatment	4-channels; 250Hz	Block design; emotional image processing task, N-back task	dPFC	After treatment, the HbO_2_ levels in the dPFC corresponding to three types of emotional images in both groups of patients increased. The HbO_2_ levels in the dPFC corresponding to sad images in the two groups of patients showed statistically significant differences at weeks 1, 2, 4, and 8
Chen, 2015 (Chengdu, China)	2015	Depressed patients *n* = 8 (3M, 5F); Age: 48.13 ± 9.75y	Depressed patients *n* = 8 (2M, 6F); Age: 47.9 ± 9.9y	Acupuncture therapy	Drug treatment	/	Task state design; emotional image processing task	dPFC	The activation level of related brain areas in the first week of acupuncture was higher than that in the Western medicine group, while the activation intensity in the Western medicine group was higher than that in the acupuncture group in the second and eighth weeks
**Insomnia**									
Xu Y. K. et al., 2022 (Beijing, China)	2022	Insomnia patients *n* = 29 (10M, 19F) Age: 18–65y	Healthy people *n* = 30 (10M, 18F) Age: 18–65y	Acupuncture therapy	/	46-channels 6.25 Hz	Emotional image processing task	Frontal lobe, Parietal lobe	The treatment of acupuncture for primary insomnia can regulate the concentration of HbO_2_ in the cerebral cortex by manipulating at specific acupoints, which can improve the function of loubus fromatis, orbitofrontal cortex and lobus parietalis related brain areas, enhance individual control of involuntary activation of extraneous stimuli, and regulate excessive arousal activity to improve insomnia
Wang et al., 2022 (Beijing, China)	2022	Young insomnia patients *n* = 10 (Age: 18–35y) Middle aged insomnia patients *n* = 12 (Age: 35–50y) Elderly insomnia patients *n* = 8 (Age: 50–65y)	/	Acupuncture therapy 3 times/week, 2 weeks	/	46-channels 6.25 Hz	Event Experimental Design	Frontal lobe	The change of HbO_2_ in the frontal lobe after acupuncture supports the traditional Chinese medicine “Yin Self-half at forty years” and the theory of insomnia that “when Yang is high, the eyes are not closed, and that when Yin is high, the eyes are closed”
**Pain disorders**									
Du et al., 2023a (Shanghai, China)	2023	Patients with the cervical-shoulder syndrome *n* = 20; Age: 36.6 ± 7.2 y	/	The E-WAA was used to administer an electrical stimulation therapy that lasted for 5 min	/	24-channels	Block design	PFC	The FP and dPFC were linked to the analgesic modulation activated by the E-WAA
Du et al., 2023b (Shanghai, China)	2023	Male patients with myofascial pain syndrome *n* = 16; Age: 47.19 ± 13.57	Male patients with myofascial pain syndrome *n* = 15; Age: 53.06 ± 17.16	Analgesic electrical stimulation combined with wrist-Ankle acupuncture	/	24-channels 10 Hz	Block design	PFC	Compared to no intervention, TENS based on wrist-ankle acupuncture can be effective in relieving pain in patients with MPS in terms of cerebral cortical hemodynamics
Shi et al., 2022 (Shanghai, China)	2022	Patients with trapezius myofascial pain syndrome *n* = 25; Age: 24–27y	Patients with trapezius myofascial pain syndrome *n* = 25; Age: 24–27y	Electronic wrist ankle needle stimulation for 5 min	Electronic wrist ankle needle stimulation for 10 s	/	Block design	PFC	E-WAA have a great analgesic effect. The FP and dPFC were relative to the analgesia neuromodulation induced by the E-WAA
**Pediatric diseases**									
Yang, 2023 (Chuzhou, China)	2023	Myopic children *n* = 118 (2M, 10F); Age: 6–14y	Healthy children *n* = 128 (2M, 10F); Age: 6–14y	Acupuncture therapy+ transcutaneous electrical acupoint stimulation therapy	/	/	Task state design; visual task	Visual cortex V1 V2	Compared to the normal vision group, the oxygenated hemoglobin in the V2 region of the myopic group during gaze movement and alternating gaze stages β Lower value
Wu, 2022 (Jinan, China)	2022	Anisometropic amblyopia children *n* = 21 (13M, 8F); Age: 6.52 ± 0.13y	Anisometropic amblyopia children *n* = 24 (13M, 11F); Age: 6.63 ± 0.13y	Transcutaneous electrical acupoint stimulation therapy	Traditional acupuncture therapy	22-channels	Event related design; visual stimulation task	Visual cortex V1 V2	After traditional acupuncture or transcutaneous electrical acupoint stimulation intervention, HbO_2_-β value in the visual cortex regions of V1 and V2 were significantly higher than before the intervention
Zhuo et al., 2022 (Xi’an, China)	2022	Attention-deficit/hyperactivity disorder children *n* = 39 (33M, 6F); Age: 8.05 ± 1.187y	Attention-deficit/hyperactivity disorder children *n* = 39 (31M, 8F); Age: 8.56 ± 1.468y	Transcutaneous electrical acupoint stimulation therapy	Sham transcutaneous electrical acupoint stimulation therapy	52-channels	Block design; visual stimulation task Go/NO go task	PFC	After treatment, the HbO_2_ in PFC of the transcutaneous acupoint electrical stimulation group increased and was higher than that of the control group
Chai, 2020 (Jinan, China)	2020	Monocular anisometropic amblyopia children *n* = 15 (7M, 8F); Age: 7–12y	Healthy children *n* = 38 (18M, 20F); Age: 7–12y	Acupuncture therapy+ conventional therapy	/	22-channels	Block design; visual stimulation task	PFC, OC	When subjected to high spatial frequency checkerboard flipping stimulation, the β-value of the visual cortex increased compared to before acupuncture, and there was no statistically significant difference at baseline
**Other disease**									
Zu et al., 2023 (Shanghai, China)	2023	Spinal cord injury patients *n* = 15 (14M, 1F) Age: 33.98 ± 6.10	Healthy people *n* = 25 (17M, 8F) Age: 25.33 ± 1.02	Aerobic exercise and wheelchair tai chi chuan	Wheelchair tai chi chuan	63-channels 10 Hz	Block design	PFC, PMC, SMA, M1	The potential and advantages of wheelchair tai chi chuan in triggering cortical muscle coupling may optimize rehabilitation after spinal cord injury
Yu et al., 2023 (Shanghai, China)	2023	Patients with bilateral tinnitus *n* = 18 (10M, 8F) Age: 27–67y	/	20-min acupuncture session every other day for a total of ten times	/	20-channels 10 Hz	The auditory stimulus paradigm	Temporal lobe	Acupuncture increased the concentration of HbO_2_ in the temporal lobe of tinnitus patients, and affected the activation of the auditory cortex

M, male; F, female; FC, functional connectivity; PFC, prefrontal cortex; dPFC, dorsolateral prefrontal cortex; vPFC, ventrolateral prefrontal cortex; FPA, frontal area; FP, frontal polar; MC, motor cortex; OC, occipital cortex; SMC, sensorimotor cortex; BA, brodmann area; S1 ± primary somatosensory cortex; M1 ± primary motor cortex; S2 ± secondary somatosensory cortex.

### 4.1 Application of fNIRS in non-drug treatment of healthy volunteers

#### 4.1.1 Tai chi chuan

Tai chi chuan originated from traditional Chinese martial arts and encompasses physical, cognitive, social, and meditative elements as multimodal mind-body exercises. Tai chi chuan has numerous cognitive benefits for young and older adults (Wayne et al., 2014; Liu et al., 2021). Moreover, many studies have used fNIRS to explore the effects of tai chi chuan training on brain function. Three studies investigated the impact of tai chi chuan on brain inhibition and control (Yang, 2019; Yang et al., 2020; Xie, 2022). The cognitive control task (flanker task) was conducted concurrently to measure and compare HbO_2_ concentrations in elderly individuals who practiced tai chi chuan regularly and those who engaged in normal daily activities. Results revealed that tai chi chuan training enhances brain inhibition and control in older adults while increasing HbO_2_ concentrations in the prefrontal lobe (Yang, 2019; Yang et al., 2020; Xie, 2022). In addition, three studies have explored the impact of tai chi chuan on brain functional connectivity. These studies demonstrated significantly higher wavelet phase coherence values, indicating the strength of functional connectivity between the prefrontal cortex (PFC), motor cortex (MC), and occipital cortex (OC) of tai chi chuan athletes compared to beginners at frequencies I, II, III, IV, and V (Xie et al., 2019; Chen W. et al., 2022; Wang et al., 2022). From a coupling direction perspective, beginners in tai chi chuan demonstrated the main coupling direction in the left brain with the right PFC, right MC (RMC), right OC, and left OC coupled toward the left MC (LMC) (Xie et al., 2019). In contrast, tai chi chuan athletes demonstrated a coupling direction from the PFC, LMC, and OC toward the RMC during rest, which varied significantly from the left-brain drive pattern observed in tai chi chuan beginners. These findings suggest changes in interactions among brain regions following long-term tai chi chuan training (Xie et al., 2019; Chen W. et al., 2022). A significant increase in functional connectivity between the left PFC and the right sensory-motor area and between the left and right sensory-motor areas was observed in long-term tai chi chuan practitioners upon transitioning from a resting state to a moving state (Wang et al., 2022). Two studies have investigated the effect of tai chi chuan on lower limb motor function. HbO_2_ concentrations were measured during the single-leg standing movements of tai chi chuan. The auxiliary motor area exhibited higher activation during the right leg kicking movement in tai chi chuan than during conventional single-leg support movement. Additionally, cerebral cortex activation is negatively correlated with postural shaking (Chen et al., 2021). Another study measured hemoglobin concentrations during negotiating obstacle with cognitive task. Tai chi chuan practice improved cognitive function in the elderly through bilateral activation of the PFC and prioritized gait performance when negotiating obstacles under dual-task conditions (Chen Y. et al., 2022). In conclusion, the study of the brain mechanism of tai chi chuan using fNIRS mainly focused on the influence of tai chi chuan on inhibitory control ability, functional connectivity of brain areas, and limb motor function. Tai chi positively impacted these functions. Tai chi exercises can activate the prefrontal lobe and increase the strength of the functional connections between the prefrontal lobe and other brain areas.

#### 4.1.2 Baduanjin

Baduanjin, a traditional Chinese healthcare movement that focuses on the “unity of form and spirit,” integrating control, breathing movements, and physical activities, plays an active role in disease prevention and rehabilitation. Jiang et al. (2019) used fNIRS to collect brain blood-oxygen signals from elderly individuals who had been exercising for a long time and those who had not practiced Baduanjin while also evaluating their cognitive function through language fluency tasks. They found that compared with elderly individuals who had not practiced Baduanjin, those who had been exercising for a long time performed better in language fluency tasks and had greater HbO_2_ concentrations in the PFC, thereby suggesting the positive impact of Baduanjin on the cognitive performance of the elderly (Jiang et al., 2019). Yao et al. (2022) found that, in addition to the Baduanjin exercise, Baduanjin imagination can also shorten the reaction time of the inhibition control task and improve the cognitive performance of the elderly (Yao et al., 2022). Moreover, Baduanjin imagination has the following advantages: (i) it is not limited by the venue, (ii) it is safe and convenient to perform, and (iii) it can be used as an alternative training method to enhance cognitive ability in the elderly. A study using the Stroop task demonstrated improved inhibitory control ability in college students after 12 weeks of Baduanjin intervention, and a correlation between the improvement in student inhibitory control ability upon Baduanjin intervention and the left dorsolateral prefrontal lobe was also identified (Li, 2020). The language fluency task and Stroop task used in the above studies were designed to assess cognitive abilities. The research results showed that both Baduanjin training and imagination can improve cognitive abilities and activate the prefrontal lobe, an important brain area for executive function, and plays a crucial role in ensuring attention allocation and enhancing cognitive control. Therefore, it is speculated that the improvement in cognition by Baduanjin may be related to the activation of the frontal lobe.

#### 4.1.3 Acupuncture

Many studies have evaluated the cortical responses of acupuncture in healthy participants using fNIRS. The block method was the mainly used study design; the evaluated parameter included *deqi* induced by acupuncture and acupuncture reinforcing and draining methods. Research on *deqi* induced by acupuncture mainly aims to observe the brain response during *deqi* to find possible evidence for the clinical treatment of diseases. Si et al. (2021) explored the effects of acupuncture *deqi* at the Hegu (LI4) point during fNIRS scanning. The results demonstrated that the bilateral prefrontal and motor cortices were significantly inhibited during acupuncture and the HbO_2_ concentration decreased. During twirling needling, the FC of the bilateral PFC increased significantly. Graph-theory analysis revealed that twirling needling improved the global efficiency of the network and reduced the length of the shortest path. Thus, acupuncture at the Hegu acupoint can regulate functional coordination between the PFC and MC. Fernandez Rojas et al. (2019) also demonstrated reduced hemodynamic response of the cerebral cortex and significant cortical activity inhibition during acupuncture at the Hegu point, which could indicate the underlying mechanism of the analgesic effect of acupuncture at the Hegu point. Song et al. (2023) demonstrated that acupuncture mainly activates the bilateral prefrontal lobes and enhances the functional connectivity between the frontal, parietal, and temporal lobes through a study of the Shousanli (LI10) acupoint, thereby indicating that the brain regions related to thinking and cognition play a role in the process of needling the Zusanli (ST36) acupoint to obtain *deqi*. Yuan et al. (2021) performed magnetic stimulation of the left temple of healthy participants while collecting the fNIRS data. The results demonstrated that PFC excitability decreased after magnetic stimulation of the Shenmen (HT7) acupoint compared with that in the resting state; moreover, the HbO_2_ concentration decreased significantly. We believe that the decrease in the blood oxygen levels confirm the sedative effect of the Shenmen acupoint. The above fNIRS research shows that acupoints have specific cortical reactions when they get deqi, and acupuncture at different acupoints has different effects on cerebral cortex hemodynamics, and acupuncture can achieve therapeutic effects through the cortical network. Hemodynamic changes and functional connections in the cerebral cortex can be used as biomarkers to quantify the regulatory effects of acupuncture.

Studies on acupuncture reinforcement and reduction have mainly focused on the differences in the brain effects attributed to various needling techniques at the same acupoint. Cao et al. (2023) recruited healthy participants to study the brain responses of three different methods of reinforcement and reduction, namely lifting-thrusting reinforcing manipulation, lifting-thrusting reducing manipulation, and reinforcing-reducing manipulation with lifting-thrusting at the Quchi (LI11) acupoint. The results revealed that different manipulations had different effects on inducing brain activation and altering the functional connectivity between the brain regions. Qu et al. (2023) recruited healthy participants to receive the “Shaoshanhuo reinforcing” and “Toutianliang reducing” acupuncture manipulations at the left Quchi point at different times (Qu et al., 2023). The results revealed that the Shaoshanhuo reinforcing method activated the bilateral primary MC and the left primary somatosensory cortex. During the Toutianliang reducing method, the bilateral PFC and primary MC were activated, and the FC between the left PFC and primary MC increased; however, the differences between the two reinforcement methods were not significant. In addition, Chen L. et al. (2023) found that even during reinforcing-reducing manipulation with the lifting-thrusting method at the Quchi point, the frontal lobe cortex of the participant and the acupuncturist revealed increased inter-brain neural synchronization, which was related to the participant’s acupuncture sensation. Different reinforcing and reducing methods at the same acupoint can have opposite effects, and the difference of cortical response displayed by fNIRS may be the central mechanism leading to different clinical effects.

### 4.2 Application of fNIRS in non-drug treatment of patients

#### 4.2.1 Cognitive dysfunction and disturbances of consciousness

Cognition is a complex process in which the brain receives and processes various types of information, such as memory, language, visual-spatial awareness, execution, calculation, understanding, and judgment. The underlying basis of cognition depends on the proper functioning of brain regions closely linked to the neural network in each brain region. Therefore, fNIRS reveals the therapeutic mechanism of TCM in treating patients with cognitive impairment by detecting changes in the HbO_2_ concentration in the cerebral cortex of patients with cognitive impairment (Eun-Sun et al., 2018; Ghafoor et al., 2019; Chen J. et al., 2020; Jeong et al., 2021; Khan et al., 2022). Six fNIRS studies were conducted in patients with cognitive impairment, specifically mild cognitive impairment (MCI) and post-stroke cognitive impairment. These studies identified effective brain regions and networks for non-drug therapies of TCM by comparing oxygenation levels and connectivity patterns between patients with cognitive impairment and healthy controls (HC) (Eun-Sun et al., 2018; Ghafoor et al., 2019; Sun, 2019; Khan et al., 2022). fNIRS can detect alterations in HbO_2_ concentrations in the frontal cortex of patients with cognitive impairment during both rest and task conditions (Ghafoor et al., 2019; Khan et al., 2022). FC analysis is frequently conducted during the resting state to examine the coordinated effects of various brain regions. Working memory tasks are often employed in the task state to examine the features of brain activation in the working memory paradigm. Specifically, researchers at Pusan National University sought to ascertain the potential of acupuncture to enhance the mental wellbeing of patients with MCI based on brain activation (Khan et al., 2022). Eleven HC and 11 MCI patients were recruited for this study. Patients received acupuncture treatment for 12 weeks, and fNIRS data were collected before and after the treatment. Prefrontal neuronal activation was measured during a working memory task. Findings demonstrated a significant decrease in PFC activation in patients with MCI compared with HC. However, acupuncture was proven to enhance the activation area and connectivity of the PFC, thereby bringing the activation area and connection map of patients with MCI closer to those of HC.

Currently, acupuncture has demonstrated a definite clinical effect in patients with consciousness disturbance; however, the mechanism underlying its effect on awakening remains unclear. Moreover, fNIRS allows for real-time monitoring of cerebral cortex activity during acupuncture, offering a theoretical foundation for the clinical use of acupuncture in patients with consciousness disturbances. Two fNIRS studies have been conducted on using acupuncture to treat consciousness disturbances (Liu et al., 2022; Xin et al., 2022). Both studies observed the effects of acupuncture on the frontal cortex activity, which plays a vital role in arousal and cognitive function. The left PFC is related to emotion, learning, and other advanced cognitive functions, while the RPFC is closely related to arousal and attention (Müri et al., 1996; Sturm and Willmes, 2001; Heekeren et al., 2006; Shin et al., 2016). The fNIRS collected data on HbO_2_ concentration during an acupuncture-specific acupoint task comprising four modules: rest, needle injection, rotation, and needle exit. The disparities between these two studies can be attributed to their respective study designs. Xin et al. (2022) conducted a self-controlled study that compared the differences in cortical activation between single acupuncture sessions and resting states. Their results demonstrated that acupuncture could activate CBF in the frontal cortex of patients with a disturbance of consciousness (Xin et al., 2022). Moreover, acupuncture enhances the strength of the cerebral cortical connections. Liu et al. (2022) conducted a randomized controlled trial to compare the functional connections in the PFC between acupuncture and sham acupuncture groups. They found that a single acupuncture session significantly enhanced resting-state functional connectivity in the dorsolateral PFC (dPFC) (Liu et al., 2022).

In conclusion, studies on the therapeutic mechanisms of acupuncture for the treatment of cognitive dysfunction and disturbances of consciousness often focus on the prefrontal region as the brain region of interest (ROI). fNIRS revealed that after acupuncture, the oxygenated hemoglobin concentration in the prefrontal lobe of the patients increased, and the functional connections in the prefrontal brain region were enhanced, thus improving the cognitive function and consciousness of the patients. The potential use of the fNIRS device to detect the effect of acupuncture in patients with cognitive dysfunction and disturbances in consciousness is significant.

#### 4.2.2 Motor dysfunction

Limb dysfunction is a common sequela of stroke, and non-drug therapies of TCM, such as acupuncture, moxibustion, and traditional exercises, are often used to treat limb dysfunction after stroke. fNIRS, with its advantages of easy operation and unrestricted space, is suitable for patients with motor dysfunction. Chen et al. recruited patients with ischemic stroke and healthy participants, and the cerebral cortex effect of the one-finger Zen manipulation at the Hegu point using fNIRS was observed. Results showed that the hemodynamics of the contralateral M1 were obviously enhanced during massage at the Hegu of the affected arm in stroke patients, but there was no similar pattern in the hemodynamics of the ipsilateral M1. There was a significant difference between the lateralization indices of the affected and healthy sides in stroke patients, indicating that the combination of Tuina and fNIRS could reflect the functional state of the sensory-motor nerve circuit (Chen Y. F. et al., 2023). It should be noted that the team conducted a randomized controlled study. The study plans to recruit 90 patients with ischemic stroke and divide them into two groups randomly. The experimental group will be given Tuina and rTMS interventions based on conventional rehabilitation, while the control group received only rTMS in addition to conventional rehabilitation. Improvements in motor function and cortical hemodynamic changes after 4 weeks of treatment will be observed (Chen Y. F. et al., 2022). Tang et al. (2022) employed fNIRS to collect and analyze changes in the functional intensity and lateralization index of patients with lower limb motor dysfunction following stroke before and after the 4-week acupuncture and rehabilitation therapy. During the data collection period, participants were required to complete four alternating tasks of 30-s walking and 30-s standing rest on a treadmill. Their results revealed that acupuncture and rehabilitation therapy can significantly improve the asymmetric activation of the lower limb motor function and sensory MC (SMC) in patients with stroke. Lower limb motor function recovery may be related to enhanced premotor cortex activation on the affected side (Tang et al., 2022). A resting-state study found that upper limb dysfunction in patients with stroke significantly improved after 2 weeks of ear acupuncture combined with routine rehabilitation treatment, and the fNIRS results demonstrated a significant increase in HbO_2_ concentrations in the primary motor area (M1) of the brain following therapeutic intervention, suggesting that activation of the M1 area of the brain may serve as a key node for ear acupuncture treatment of upper limb dysfunction (Zhang et al., 2023). Another study used fNIRS to explore the mechanism of action of tai chi chuan imagination exercises. The study revealed that the tai chi chuan imagination exercise group displayed a higher HbO_2_ concentration in the SMC than the simple imagination exercise and conventional treatment groups. This indicates that tai chi chuan imagination exercise can effectively activate the corresponding cerebral MC, thereby offering novel insights into optimizing stroke treatments (Luo et al., 2022). The above research found that the improvement in limb dysfunction was related to the activation of the primary sensorimotor cortex, auxiliary motor area, premotor cortex, and other brain areas, which provides a scientific basis for the neural mechanism of acupuncture and moxibustion, tai chi chuan, and other therapies to improve limb dysfunction after stroke.

Parkinson’s disease (PD) is a common neurodegenerative disorder with motor symptoms such as tremor, stiffness, bradykinesia, and postural instability, impairing patient mobility and resulting in limited independence (Balestrino and Schapira, 2020). Some studies have demonstrated that acupuncture can increase the stride length and swing times of patients with PD and reduce the rhythm that cannot be improved by levodopa treatment; moreover, acupuncture improves balance control by activating the auxiliary motor area and PFC as demonstrated by fNIRS, thereby improving gait disorders in patients with PD. Therefore, acupuncture is considered to be an effective adjuvant treatment for the PD (Jang et al., 2020).

#### 4.2.3 Mental and behavioral disorders

The PFC, which is involved in cognitive processing and emotional regulation (Dunlop et al., 2017), elucidates cognitive and emotional symptoms associated with depression and anxiety. Acupuncture is a safe and effective treatment option for depression and anxiety. Several researchers have hypothesized that the therapeutic effects of acupuncture on depression can be partially attributed to the modulation of the PFC. To gain a better understanding of the antidepressant effects of acupuncture, four studies used fNIRS to examine the prefrontal lobe activation and functional connectivity in patients with depression who received acupuncture. Commonly used task paradigms include emotional picture processing (Chen, 2015), working memory (Miao, 2016), and speech-fluency tasks (Zhang et al., 2021). Zhang et al. (2021) obtained fNIRS data from patients with severe depression who underwent speech fluency and Baihui acupoint tasks to assess PFC activation during acupuncture at the Baihui acupoint. These findings suggest that a single acupuncture session may increase frontopolar cortex activation in patients with severe depression. A correlation was noted between the left dorsolateral PFC activation during executive functioning through acupuncture and the severity of depressive symptoms in patients diagnosed with severe depression (Zhang et al., 2021). Furthermore, studies have analyzed the functional connectivity of the dorsolateral prefrontal lobe by collecting resting-state data before and after acupuncture in patients with depression (Wong et al., 2021). One study demonstrated a significant temporal correlation in functional connectivity between channel pairs in the dPFC during the resting state. Patients diagnosed with anxiety disorders exhibit inadequate prefrontal lobe activation during emotion regulation. Another fNIRS study investigated the immediate effect of acupuncture on the left dPFC function in patients with generalized anxiety disorders (Xiang, 2017). Acupuncture activates the left dPFC in individuals with generalized anxiety disorders, as evidenced by an increase in HbO_2_ and a decrease in dHb concentrations. However, this finding was not observed in healthy individuals. Thus, emotion regulation by acupuncture in generalized anxiety disorders may be associated with the activation of the left dPFC. The above research shows that the central mechanism of acupuncture in improving depression and anxiety symptoms may be related to increased HbO_2_ levels in the dorsolateral prefrontal lobe and that fNIRS has great potential in the application of disease severity and evaluation of curative effects.

#### 4.2.4 Insomnia

Insomnia is a prevalent neurological disorder that can adversely affect patients’ emotions, cognition, and mental states (Perez and Salas, 2020). To validate the efficacy of non-drug therapies of TCM for insomnia, several researchers have employed fNIRS to monitor brain function in patients with insomnia and have thus generated objective evidence for the application of non-drug therapies of TCM to further promote their clinical application. Xu Y. K. et al. (2022) used fNIRS to detect the effect of acupuncture treatment on changes in cerebral HbO_2_ concentration during emotional face recognition by patients with primary insomnia. They found that acupuncture improved the function of the frontal, orbitofrontal cortex, and parietal-related brain regions by regulating the HbO_2_ concentration in the cerebral cortex, thereby enhancing the individual’s control of involuntary activation of irrelevant stimuli and adjusting the state of hyperarousal to improve insomnia (Xu Y. K. et al., 2022). Moreover, acupuncture demonstrated different effects on the HbO_2_ concentrations in the frontal lobe of patients with insomnia of varying ages under the emotional face conditions of “yin” and “yang” attributes. This verified the rise and fall of “yin” and “yang” as the reason for the varied effects of acupuncture treatment on insomniac patients of different ages (Wang et al., 2022). These two studies show that acupuncture can regulate the concentration of HbO_2_ in the frontal lobe of patients with insomnia to reduce hypersensitivity and further explore the neural mechanism of acupuncture in the treatment of insomnia. However, because brain development and degeneration at different ages affect the concentration of HbO_2_ in the brain, the effect of acupuncture on HbO_2_ in specific brain regions needs to be further studied.

#### 4.2.5 Pain disorders

Pain is a subjective condition involving sensory, emotional, cognitive, and other factors; thus, relying only on a patient’s subjective report for pain assessment and efficacy judgment is unreliable. Pain activates corresponding areas of the cerebral cortex, and it is important to objectively assess pain by detecting cortical activation using fNIRS. Electronic wrist and ankle acupuncture bracelets (E-WAAs) combine the advantages of wrist and ankle acupuncture with percutaneous electrical nerve stimulation and have demonstrated good acute and chronic pain management. Some scholars have studied the analgesic mechanism of E-WAA through fNIRS and found that frontopolar area (FPA) and dPFC are significantly involved in pain processes and regulation; moreover, decreased blood oxygen concentration in the FPA and dPFC brain regions was related to the analgesic regulation of E-WAA (Shi et al., 2022; Du et al., 2023a,b). Thus, quantitative analysis of blood oxygen concentration may be considered a potential method for more accurately identifying the analgesic effects of E-WAA and its mechanisms.

#### 4.2.6 Pediatric diseases

Functional near-infrared spectroscopy is commonly used to investigate the pathogenesis of the cerebral visual cortex and the therapeutic mechanisms of acupuncture in pediatric diseases, such as childhood myopia, amblyopia, and attention-deficit hyperactivity disorder. Typically, HbO_2_ signals are detected in the V1 and V2 visual cortices of children with myopia and amblyopia, and activation levels are assessed using a visual task paradigm. In some studies, the HbO_2_ β concentration in the V2 region of children with myopia was notably lower than in healthy children (Yang, 2023), while the V1 and V2 regions of children with amblyopia exhibited noticeably lower HbO_2_ β levels as compared to those of healthy children (Wu, 2022). In children, amblyopia is closely associated with functional changes in the primary and secondary visual cortices. In addition, HbO_2_ content in the ipsilateral visual cortex was lower than that in the contralateral visual cortex. This asymmetry in bilateral visual cortex involvement explains alterations in brain function in children with anisometropic amblyopia. Acupuncture, a widely used method to treat children with near-amblyopia, involves acupuncture with less pain (Chai, 2020), transcutaneous acupoint electrical stimulation, and traditional acupuncture (Wu, 2022; Yang, 2023). fNIRS was used to measure HbO_2_ levels before, during, and after the intervention. Statistical analysis revealed differences in the acupuncture regulation mechanisms. These findings demonstrate that acupuncture can enhance blood oxygen activity in the visual cortex and increase HbO_2_ content in areas V1 and V2 (Chai, 2020; Wu, 2022). Researchers have used fNIRS to examine brain responses in children with attention-deficit/hyperactivity disorders treated with percutaneous acupoint electrical stimulation (Zhuo et al., 2022). The HbO_2_ concentration was measured using a go/no-go task before and after treatment. The HbO_2_ concentration in the prefrontal lobe was higher after percutaneous acupoint electrical stimulation than that in the sham stimulation group, indicating a sensitive response area to percutaneous acupoint electrical stimulation. What deserves our attention is the accessibility of fNIRS and its ability to provide valuable results during physical exercise, making it more suitable for evaluating brain function in infants and young children.

#### 4.2.7 Other diseases

In addition, fNIRS has been preliminarily applied to diseases such as spinal cord injury and tinnitus. Spinal cord injury is the most serious complication of spinal-related diseases, often resulting in lower limb dysfunction and wheelchair dependency. Wheelchair tai chi chuan, an emerging practice suitable for patients with spinal cord injury, has demonstrated improved motor function and neurological recovery (Qi et al., 2018). Zu et al. (2023) performed an fNIRS analysis in patients with spinal cord injury and healthy individuals while performing wheelchair tai chi chuan. Their results demonstrated that the bilateral prefrontal lobes, primary motor areas, and premotor area of the brain of patients with spinal cord injury were more greatly activated than those of the healthy participants during the wheelchair tai chi chuan exercise, indicating that patients with spinal cord injury needed more mental activities to complete the activities of the upper limb muscles. Thus, wheelchair tai chi chuan may compensate for the reduction in cortical-muscular coupling through muscle activation (Zu et al., 2023). Tinnitus, which is the auditory phantom perception of a ringing, hissing, or buzzing sound in the absence of an objective physical sound source, is associated with lesions of the central nervous system (Baguley et al., 2013; Dalrymple et al., 2021). Yu et al. (2023) treated patients with bilateral tinnitus using performed fNIRS measurements before and after treatment using the block paradigm of the auditory stimulus design. The results revealed that the patients’ scores on the hearing impairment-related scale were reduced after acupuncture treatment; moreover, the HbO_2_ concentration in the temporal auditory cortex was increased and the change in HbO_2_ concentration was significantly correlated with the score of hearing impairment-related scales. This indicates that acupuncture can activate the auditory cortex to play a therapeutic role (Yu et al., 2023).

## 5 Discussion

This article is so far the first review on the application of fNIRS in non-drug therapies of TCM. In this review included 46 articles describing the effects of acupuncture, massage, Taijiquan, and Baduanjin on the brain. Although we searched for research in many countries worldwide, most research has been carried out in China, and a few studies have been conducted in South Korea and Australia. A total of 11 randomized controlled studies were included, and the subjects of the remaining studies were generally compared between patients and healthy subjects. This design was related to the purpose of the fNIRS study. One is to explore the difference of cerebral hemodynamics between a disease and healthy people, and to clarify the effect of the disease on brain function. Another is to provide a reference for the treatment of patients, that is, healthy human brain function as the baseline state, and take this as the goal of affected brain function recovery.

The fNIRS analysis revealed specific cortical responses in areas commonly associated with these therapies, such as the dPFC, sensorimotor area, and supplementary motor area. Non-drug therapies of TCM can alter blood oxygen concentration levels in specific regions of the cerebral cortex, thus resulting in “activation.” Several TCM interventions can result in different degrees of “activation.” For instance, practicing tai chi chuan and Baduanjin can increase the HbO_2_ concentration in the prefrontal lobe, thereby enhancing cognitive, executive, and control functions. Contrastingly, certain acupuncture methods can lead to increased or decreased HbO_2_ concentrations, eliciting excitatory or inhibitory effects. Furthermore, non-drug therapies of TCM can improve functional connections in certain brain regions and regulate the coordination of different regions, resulting in the integration of the cortical brain network. This intervention can enhance recovery from sports injuries, cognitive impairment, and negative emotional states and ameliorate pain responses. Modification of brain activity or functional connectivity intensity between regions provides compelling evidence for elucidating the central mechanism of non-drug therapies of TCM. Research on fNIRS related to healthy people mainly includes two aspects. The first is to explore the cortical central effect of tai chi chuan and Baduanjin in enhancing control and improving cognitive function for a long time, mainly the activation of the dorsolateral prefrontal lobe, and enhancing the functional connection with motor area and occipital lobe. The second is to observe the hemodynamic changes of acupuncture at specific points. fNIRS research shows that specific hemodynamic changes only occur when acupuncture gets *deqi*, and acupuncture at points can produce a negative activation effect and reduce cortical excitability, which may be the neural basis for relieving pain, insomnia, and other diseases. There are dissimilar reports on hemodynamic changes caused by different manipulations of acupuncture at the same point, which need further study. The patient-related fNIRS research mainly focuses on the improvement of symptoms or functions of cognitive dysfunction, motor dysfunction, mental and behavioral disorders, insomnia, pain, pediatric diseases and other diseases by non-drug therapies of TCM, and the mechanism of action of the cerebral cortex. The above research shows that non-drug therapies of TCM can increase blood oxygen concentration levels in the prefrontal lobe, regulate functional connection between the prefrontal lobe and the motor area and occipital lobe, improve cognitive function in patients with cognitive impairment, and relieve negative symptoms of depression and anxiety disorders. The decrease of oxygen concentration in frontal pole and dorsolateral prefrontal lobe may be one of the mechanisms to improve insomnia. Non-drug therapies of TCM can activate primary motor area and auxiliary motor area, which is helpful to improve limb motor function and balance ability of patients with hemiplegia and Parkinson’s disease.

In future studies, investigating the interplay between various factors, including treatment, treatment frequency, and stimulation intensity, with brain region activation and brain network changes is necessary to enhance our understanding of the underlying mechanisms of non-drug therapies of TCM. To obtain more precise test results, the experimental protocol, number of probes, placement positions, fNIRS data acquisition, and processing methods should be standardized. Furthermore, broadening the scope of disease spectra for clinical applications of fNIRS and investigating the mechanism of action of other non-drug therapies of TCM, such as Wuqinxi, Yijinjing, and Guasha, are recommended. This will offer a more scientific, comprehensive, and standardized basis for the clinical application of non-drug therapies of TCM in the future. In summary, fNIRS, a rapidly developing brain imaging technique, has greatly contributed to elucidating the mechanism of action of non-drug therapies of TCM. This technology is anticipated to become even more instrumental in clinical diagnosis, treatment, and evaluation in the future.

## 6 Limitation

Currently, fNIRS is largely employed in studies on non-drug therapies of TCM to investigate the underlying mechanisms of action in healthy participants and the therapeutic effects in patients. fNIRS is used as a prediction method to dynamically monitor changes in hemoglobin content in the brain in real-time using a specific paradigm to determine whether it could be a potential target for healthcare or treatment. However, this review has certain limitations. First, the age range of the participants included in the study was large, with no unified standard, and brain function was not constant, especially as it changed with age (Damoiseaux, 2017). Thus, the impact of changes in the physiological state on the results of this study is unknown. Second, the sample size in most studies was approximately 10–30 patients; thus, increasing the sample size would have provided more reliable results. Third, during the resting-state measurement, the participants’ thoughts could have caused disturbances, resulting in changes in brain signals. Therefore, the objectivity of the collected data should be ensured. Finally, because of the small number of RCTs included, there is a potential risk of bias. Therefore, strict RCT should be included in future studies to elaborate on the clinical application of fNIRS in non-drug therapies of TCM.

## 7 Conclusion

In this review, we introduce the principle of fNIRS and its application in non-drug therapies of TCM. Although fNIRS cannot be directly used for diagnosis, it can reflect the inhibitory control ability, cognitive ability, etc., of subjects through relevant task paradigms, and brain function changes caused by non-drug therapies of TCM through blood oxygen levels to reveal objective neural mechanisms. However, there are few randomized controlled studies in the existing literature, and there is a potential risk of bias.

## Author contributions

SF: Writing—original draft. FL: Writing—original draft. XZ: Writing—original draft. YW: Writing—original draft. YL: Writing—original draft. HC: Writing—original draft. ML: Writing—review and editing. YW: Writing—review and editing.
